# Correlations in Full-Body Postural Morphology: A Cross-Sectional Exploratory Pilot Study Using a Dual-Camera Structured Light System

**DOI:** 10.3390/diagnostics16152352

**Published:** 2026-07-27

**Authors:** Giuseppe Messina, Francesca Campoli, Omar Gaetano Maria Mingrino, Patrik Drid, Jessica Brusa, Dorota Kostrzewa-Nowak, Donatella Di Corrado, Elvira Padua, Angelo Iovane, Vincenzo Cristian Francavilla

**Affiliations:** 1 Department of Human Sciences and Promotion of the Quality of Life, San Raffaele University, 00166 Rome, Italy; francesca.campoli@uniroma5.it (F.C.);; 2 Faculty of Sport and Physical Education, University of Novi Sad, 21000 Novi Sad, Serbia; 3 Department of Clinical and Molecular Biochemistry, Pomeranian Medical University, 70-204 Szczecin, Poland; dorota.kostrzewa.nowak@pum.edu.pl; 4 Department of Sport Sciences, Kore University, Cittadella Universitaria, 94100 Enna, Italy; 5 Sport and Exercise Sciences Research Unit, Department of Psychology, Educational Science and Human Movement, University of Palermo, 90128 Palermo, Italy

**Keywords:** posture, rasterstereography, structured light, sagittal balance, spine, non-invasive, Spine3D, technical note

## Abstract

Postural assessment is fundamental for the diagnosis and monitoring of musculoskeletal disorders. While single-camera structured light systems exist, they often lack the capability for simultaneous full-body capture, potentially leading to motion artifacts and incomplete analysis of the postural chain. This technical note utilizes a dual-camera architecture to explore the clinical feasibility and identify postural correlations in a healthy male sample. A cross-sectional exploratory study was conducted on 44 healthy male subjects (age 25–65 years). Posture was assessed using a Spine3D system equipped with two Time-of-Flight (ToF) cameras for instantaneous, radiation-free acquisition. Descriptive statistics and a Pearson correlation analysis (with FDR correction) were calculated for 38 postural parameters. Data normality was verified, and effect sizes were interpreted according to standard thresholds. The analysis identified that approximately 21% (148 out of 703 possible pairs) of correlations were statistically significant (p_FDR < 0.05). Among the most clinically meaningful were a strong positive correlation between cervical and lumbar lordosis (r = +0.666) and a moderate negative correlation between cervical lordosis depth and craniovertebral angle (r = −0.465), indicating that a more pronounced cervical curve is associated with a more protruded head posture. The dual-camera structured light system demonstrated potential clinical utility for a global and rapid postural assessment. The data confirm the close interdependence between various body segments, emphasizing the importance of a holistic approach. However, as an exploratory technical note, future rigorous studies are needed to establish reliability and validate these findings against radiographic gold standards.

## 1. Introduction

The assessment of posture and spinal morphology is a cornerstone in the diagnosis and monitoring of numerous musculoskeletal disorders, whose impact on quality of life is widely documented [1,2]. Physiological aging induces progressive changes in the spine, including reduced bone mass and degenerative changes in discs, facet joints, and ligaments [3,4]. These alterations can compromise spinal biomechanics, affecting posture and load-bearing capacity [5].

Traditionally, the analysis of spinal curves has relied on radiographic imaging (X-ray), which, despite being the gold standard for measuring bone angles [6], exposes the patient to ionizing radiation. This exposure, a known and documented risk [7,8], severely limits the repeatability of assessments, making radiographs an unsuitable tool for longitudinal screenings, frequent monitoring of deformity progression, or analysis in sensitive populations [9].

In recent years, the importance of sagittal balance has been widely recognized as a critical factor for spinal health and quality of life, with significant links to pain, function, and disability [10,11]. Consequently, the need for alternative diagnostic tools has grown. Non-invasive structured light technologies, such as rasterstereography, have emerged as a safe and reliable solution [12,13]. These systems allow for a 3D reconstruction of the trunk surface, from which clinical parameters related to the underlying spinal morphology can be derived, as demonstrated by numerous intra- and inter-operator reliability studies [14,15,16].

However, a significant gap remains in the current application of these technologies. Many existing systems rely on a single camera architecture, which often requires multiple scans or repositioning to capture the entire body, from the cervical spine to the lower limbs. This limitation can introduce motion artifacts and prevents a truly simultaneous, holistic evaluation of the entire postural chain.

Therefore, the objective of this technical note is to explore the clinical feasibility and identify postural correlations in a healthy male sample using a novel dual-camera structured light system. By capturing the entire body instantaneously, this study aims to provide preliminary normative data and investigate the interdependencies between different anatomical regions, particularly focusing on the cervical spine and lower limbs. This manuscript is presented as a technical note to introduce the technological advancement and its exploratory correlational capabilities, acknowledging that full clinical validation is beyond its current scope.

## 2. Materials and Methods

### 2.1. Study Design and Sample

A cross-sectional exploratory pilot study was conducted to characterize postural parameters in a convenience sample. A total of 44 male subjects were recruited, with an age range of 25 to 65 years (mean age: 45 years). The sample size was determined based on feasibility for an initial exploratory analysis of the new dual-camera system, rather than a formal power calculation for effect size estimation.

The selection of an exclusively male sample at this stage was dictated by the need to optimize data acquisition, which requires the removal of the bra for a correct visualization of the dorsal anatomical landmarks, in line with standard protocols for back surface analysis [17]. Furthermore, focusing on a single gender reduces confounding variables related to known sex-based differences in spinal morphology during this initial exploratory phase.

### 2.2. Inclusion and Exclusion Criteria

Inclusion criteria were being male and having a chronological age within the 25–65-year range. Participants were required to have a moderate physical activity level and a normal BMI range to ensure a relatively homogeneous healthy baseline musculoskeletal status.

The following exclusion criteria were applied:

1. Positive history of traumatic events affecting the musculoskeletal system in the last 6 months.
2. Current use of orthodontic appliances, bites, or other intra-oral devices.
3. Use of hearing aids.

### 2.3. Instrumentation: Dual-Camera System

Postural assessments were performed using a structured light system (Spine3D, Sensor Medica, Guidonia Montecelio, Rome, Italy), a non-invasive device utilized in previous studies for postural analysis [18]. The metrological qualities, reliability, and validation of the instrument’s AI algorithms and measurements have been documented in the recent literature [19,20,21]. The system features a fixed architecture of two VZense DS86 cameras (see Table 1).

The hardware configuration includes the following:

- Upper Camera: Positioned at 1365 mm from the ground, dedicated to acquiring the upper trunk and head.
- Lower Camera: Positioned at 600 mm from the ground, dedicated to acquiring the pelvis and lower limbs.

This arrangement ensures an instantaneous full-body acquisition (from head to heels) with a single shot, minimizing possible patient motion artifacts. Specific solutions were implemented for managing interference between the ToF sensors (temporal desynchronization) and for aligning the two-point clouds into a single 3D mesh (Iterative Closest Point, ICP, algorithm).

### 2.4. Landmark Identification and Biomechanical Parameters

The system’s analysis is based on the identification of specific anatomical landmarks. The software integrates an automatic recognition system based on Artificial Intelligence, which uses a deep neural network trained on a dataset of over 3000 images manually annotated by expert operators. The validation dataset consisted of 500 independent images, representing a diverse demographic (age 18–75, both sexes, varying BMI). The AI model achieved a mean absolute error (MAE) of 2.1 mm and a root mean square error (RMSE) of 2.8 mm in landmark localization compared to expert manual annotation, demonstrating high accuracy and low measurement uncertainty [19].

The full-body acquisition allowed for the calculation of various clinical parameters:

- Advanced Cervical Spine Analysis: Calculation of the Cranio-Vertebral Angle (estimation of head protrusion), Lateral Head Tilt, and Head Rotation on the transverse plane.
- Lower Limb Analysis: Measurement of pelvic discrepancy and tilt, knee valgus/varus and flexion/extension angles (coronal and sagittal planes), and morphometric length of femur and tibia.

### 2.5. Acquisition Protocol

All measurements were performed in an environment with standardized lighting conditions. Participants were asked to assume a natural, relaxed orthostatic position, barefoot and wearing only underwear (briefs). Subjects wearing prescription glasses were asked to keep them on. The acquisition was initiated by an expert operator with a single command.

### 2.6. Statistical Analysis

Data analysis was performed using the Python 3.10 programming language and the Matplotlib and SciPy scientific libraries. Descriptive statistics (mean and standard deviation) were calculated for the main postural parameters. The normality of the data distribution was verified using the Shapiro–Wilk test, confirming that parametric tests were appropriate.

To investigate the relationships between variables, an exploratory correlation analysis was conducted. The Pearson correlation coefficient (r) was calculated for all pairs of parameters. Effect sizes were interpreted according to standard thresholds: |r| < 0.3 weak, 0.3 ≤ |r| < 0.5 moderate, and |r| ≥ 0.5 strong. To manage the problem of multiple comparisons (703 possible pairwise correlations for 38 variables), the False Discovery Rate (FDR) correction according to the Benjamini–Hochberg method was applied. A correlation was considered statistically significant if the corrected *p*-value (p_FDR) was less than 0.05. Given the large number of comparisons, the analysis remains strictly exploratory.

## 3. Results

### 3.1. Descriptive Statistics

**Figure 1:**
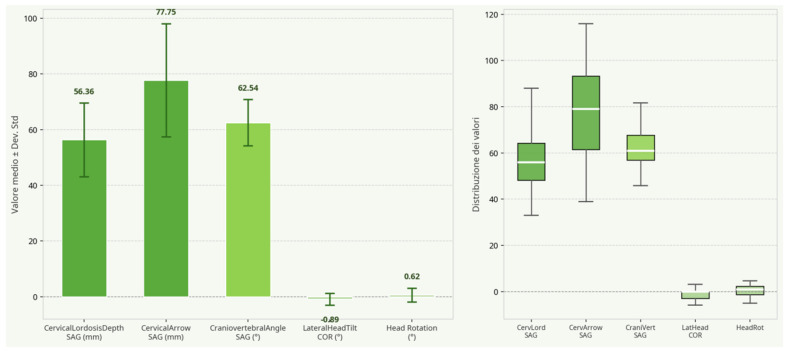
Descriptive statistics of the main cervical parameters. The left panel shows the mean and standard deviation for each parameter. The right panel shows the boxplot distribution for each parameter.

The descriptive analysis of the main postural parameters of the cervical spine and the coronal plane is summarized in the Table 2. All parameters were calculated on a sample of *N* = 44 subjects. These values represent preliminary data for this specific cohort and should not be interpreted as established normative reference values (see Figure 1).

### 3.2. Correlation Analysis

#### 3.2.1. Correlation Between Cervical Lordosis and Craniovertebral Angle

**Figure 2:**
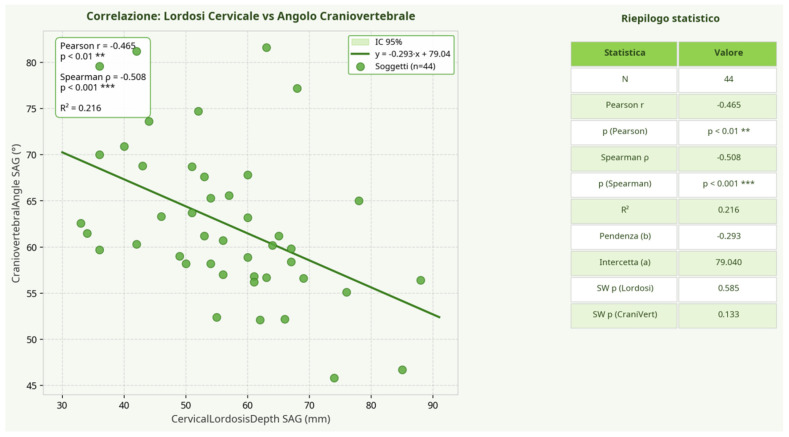
Scatter plot showing the negative correlation between cervical lordosis depth (mm) and craniovertebral angle (°). The line represents the linear regression line, with the 95% confidence interval shown in light green. ** indicates p<0.01; *** indicates p<0.001.

The specific analysis between the depth of the cervical lordosis (CervicalLordosisDepth_SAG) and the craniovertebral angle (CraniovertebralAngle_SAG) revealed a negative, statistically significant, and moderate correlation (r = −0.465; *p* = 0.0015). This result suggests that as the depth of the cervical lordosis increases, the craniovertebral angle tends to decrease, indicating a potential association between a more pronounced cervical curve and a more protruded head posture (Figure 2).

#### 3.2.2. Global Correlation Matrix and Clinically Relevant Relationships

**Figure 3:**
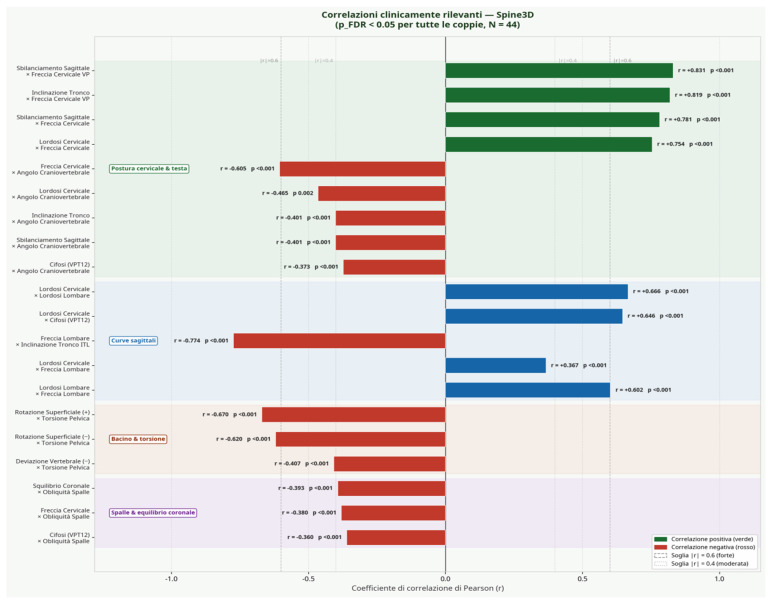
Bar chart of the main clinically relevant correlations, grouped by anatomical area. Green and blue bars indicate a positive correlation; red bars a negative correlation. All pairs shown are statistically significant (p_FDR < 0.05).

The correlation analysis, extended to 38 postural parameters, identified that approximately 21% (148 out of 703 possible pairs) exhibited a statistically significant correlation (p_FDR < 0.05 and |r| ≥ 0.3) after correction for multiple comparisons. Figure 3 graphically summarizes the most clinically relevant correlations, grouped by anatomical domain.

The main results can be summarized as follows:

- Cervical Posture and Head Domain: A strong positive correlation is observed between the sagittal imbalance of the trunk and head protrusion (measured as CervicalArrow_VP, r = +0.831). The craniovertebral angle, as already seen, is negatively correlated not only with cervical lordosis (r = −0.465) but also with the cervical arrow (r = −0.605), confirming that head posture is closely linked to the morphology of the entire cervical and thoracic spine.
- Sagittal Curves Domain: A strong positive co-variation emerges between cervical and lumbar lordosis (r = +0.666), supporting the hypothesis of a functional coupling between the two curves. A positive correlation is also noted between cervical lordosis and the angle of thoracic kyphosis (r = +0.646).
- Pelvis and Torsion Domain: The surface rotation of the trunk was found to be negatively correlated with pelvic torsion (r up to −0.670), suggesting a possible compensatory mechanism between the two body districts on the transverse plane.

## 4. Discussion

This technical note explored the clinical feasibility of a dual-camera structured light system for a global postural assessment. The architecture and analysis algorithms allowed for the acquisition of a complex dataset, from which numerous clinically relevant correlations emerged, in line with the literature investigating postural interdependencies [22,23].

The data confirm the close interconnection between the various body districts. The strong positive correlation between cervical and lumbar lordosis (r = +0.666) supports the concept of functional coupling between the spinal curves, a concept discussed in the literature [24,25]. Head posture, in particular, is not an isolated phenomenon but appears to be the result of a complex postural chain, being significantly correlated with the morphology of the sagittal curves of the trunk and the overall balance of the patient [11,26].

It is important to distinguish the morphological surface topography utilized in this study from traditional posturography. While posturography primarily assesses balance control and stability by measuring the center of pressure sway on a force platform, the present system focuses on the 3D morphological reconstruction of the spine and body surface. These two approaches are complementary; one evaluates the dynamic control of posture, while the other provides a static, structural assessment of the postural chain.

The main strength of this study lies in the use of a technology that overcomes some limitations of traditional methods. The non-invasive and radiation-free nature of the system suggests potential clinical utility for applications where repeated assessments are necessary, such as monitoring the progression of a deformity or evaluating the effectiveness of a rehabilitation intervention [27,28,29]. Furthermore, the instantaneous acquisition minimizes motion artifacts.

However, as a technical note presenting preliminary exploratory data, this study has several major methodological limitations that must be explicitly acknowledged: 1. Lack of Validation: The most critical limitation is the absence of concurrent validity testing against a gold standard, such as radiographic imaging (X-ray) or MRI. The absolute accuracy of the measurements provided by this specific dual-camera configuration remains to be established. 2. Absence of Reliability Analysis: No intra-day or inter-day reliability (test–retest) analysis was performed. Measurement error considerations are therefore absent, which limits the emphatic interpretation of the data. 3. Exploratory Nature: The correlation analysis, despite FDR correction, remains strictly exploratory. The large number of variables (38) compared to the sample size increases the risk of spurious findings. 4. Sample Limitations: The sample size is small (N = 44) and restricted to healthy adult males. This limits the generalizability of the findings to females, different age groups, or clinical populations with specific spinal pathologies.

## 5. Conclusions

In conclusion, this technical note demonstrated the feasibility of using a dual-camera structured light system to identify significant correlations in full-body postural morphology. The results highlight the complex interrelationships between different body segments, reinforcing the need for a holistic approach in postural assessment. While the system shows potential clinical utility due to its non-invasive nature, it is not yet ready for definitive clinical application. The findings presented here are strictly exploratory. Substantial methodological improvements, including rigorous reliability analysis and validation against established radiographic gold standards, are mandatory before this system can be fully endorsed for clinical practice.

## Figures and Tables

**Table 1:** Technical specifications of the cameras used in the system.

Technical Feature	System Specifications (VZense DS86)
RGB Camera Resolution	1600 × 1200 pixels @ 30 fps
ToF Camera Resolution	640 × 480 pixels @ 15 fps
Operating Range	0.15–5 m
Accuracy Error	<1% at 1 m distance
Data Connection	Gigabit Ethernet

**Table 2:** Descriptive statistics of the main cervical and coronal parameters.

Parameter	Unit	Mean	Std. Dev.	Min	Max
CervicalLordosisDepth_SAG	mm	56.36	13.25	33.0	88.0
CervicalArrow_SAG	mm	77.75	20.36	39.0	116.0
CraniovertebralAngle_SAG	°	62.54	8.35	45.8	81.6
LateralHeadTilt_COR	°	−0.89	2.12	−5.8	3.2
Head Rotation	°	0.62	2.47	−5.0	4.7

## Data Availability

The original contributions presented in this study are included in the article. Further inquiries can be directed to the corresponding authors.
